# Retraction Note: LRPPRC regulates malignant behaviors, protects mitochondrial homeostasis, mitochondrial function in osteosarcoma and derived cancer stem-like cells

**DOI:** 10.1186/s12885-025-13492-7

**Published:** 2025-01-14

**Authors:** Ziyi Zhao, Yingwei Sun, Jing Tang, Yuting Yang, Xiaochao Xu

**Affiliations:** 1 TCM Regulating Metabolic Diseases Key Laboratory of Sichuan Province, Hospital of Chengdu University of Traditional Chinese Medicine, Chengdu, 610041 People’s Republic of China; 2 Department of Pharmacy, Hospital of Chengdu University of Traditional Chinese Medicine, Chengdu, 610041 People’s Republic of China; 3 Chongqing Three Gorges Medical College, Chongqing, 404120 People’s Republic of China; 4 College of Food and Biological Engineering, Chengdu University, Chengdu, 610000 People’s Republic of China

**Correction: BMC Cancer 23, 935 (2023)**

**https://doi.org/10.1186/s12885-023-11443-8**

The Editor has retracted this article because overlap has been found in the figures and raw data with the following publications [1–3], specifically:

A panel in Fig. 2d appears to overlap with a panel in Fig. 2.1 in [1]. In the flow cytometry plot for MG63 CSCs shLRPPRC + Mock in Fig. 5A appears to overlap with the plot for U2OS-CSCs 200 nmol/L DOX in Fig. 4B [3]. Similarly, the flow cytometry plot for MG63 CSCs shLRPPRC + carboplatin in Fig. 5A appears to overlap with the plot for MG-63 CSCs 100 µmol/L MXT & 15 µmol/ CHE in Fig. 4B [3]. Original images supplied by the authors in support of their article appear to overlap with panels in papers [1] and [2], by different authors. Further similarities and apparent displacement was found in the images supplied by the authors for the replicates in Fig. 2C. Finally, an author appears to have a potential conflict of interest with Shanghai YuanPing biomedical ltd. that was not declared before publication. The Editor therefore no longer has confidence in the reliability of underlying data, results and conclusions of this article.

The authors have not responded to correspondence regarding this retraction.

## References

[1] Zhang Cuiwei, Li Shuiqin, Zhao Ziyi. β-Elemene Promotes Apoptosis Induced by Hyperthermia via Inhibiting HSP70. Dis Markers. 2022;2022(7313026):10. 10.1155/2022/7313026. 10.1155/2022/7313026 PMC9325567 35903296

[2] BioMed Research International. Retracted: N-myc Downstream-Regulated Gene 1 (NDRG1) Regulates Vascular Endothelial Growth Factor A (VEGFA) and Malignancies in Glioblastoma Multiforme (GBM). BioMed Res Int. 2023;2023(9794063):1. 10.1155/2023/9794063. 10.1155/2023/9794063 PMC10567427 37829051

[3] Chen Zhixing, Yang Hui, Zhang Qianlu, Qiongying Hu, Zhao Ziyi. Chelerythrine Inhibits Stemness of Cancer Stem-Like Cells of Osteosarcoma and PI3K/AKT/mTOR Signal. J Oncol. 2022;2022(6435431):11. 10.1155/2022/6435431. 10.1155/2022/6435431 PMC9484924 36131794

